# Isotretinoin-induced hair growth in a case of hereditary hypotrichosis simplex of the scalp: A promising therapeutic approach

**DOI:** 10.1016/j.jdcr.2024.06.020

**Published:** 2024-07-05

**Authors:** Almuntsrbellah AlMudimeegh, Khalid Nabil Nagshabandi

**Affiliations:** Department of Dermatology, College of Medicine, King Saud University, Riyadh, Saudi Arabia

**Keywords:** congenital hypotrichosis, hereditary hypotrichosis, HHS, isotretinoin, vitamin A derivative

## Introduction

Hereditary hypotrichosis simplex (HHS) is a rare and uncommon genodermatosis characterized by miniaturization of the hair follicle.^1^ Patients commonly present with concerns regarding insufficient hair length and density. HHS can manifest solely on the scalp or may also involve other areas of body hair. Diagnosis typically relies on the exclusion of other congenital hair and ectodermal disorders.^1^ Historically, effective treatment options for this condition have been lacking. Despite limited treatment options, recent studies have shown promising outcomes with interventions such as topical, sublingual, and oral minoxidil, topical gentamicin, and platelet-rich plasma injection combined with topical minoxidil 2%.^2^, ^3^, ^4^, ^5^, ^6^

Isotretinoin, a synthetic derivative of vitamin A, targets various pathogenic factors involved in sebum production suppression and acne formation. It is commonly employed in the management of moderate-to-severe acne vulgaris.^6^ Despite the known mucocutaneous side effects of isotretinoin, including telogen effluvium, thinning hair, and hair loss.^7^^,^^8^ In a rare and intriguing instance, we report a patient with HHS of the scalp that exhibited signs of hair growth after starting isotretinoin therapy. Which to the best of our knowledge, is the first of its kind to link hair growth and improvement in association with the use of isotretinoin.

## Case report

**Fig 1:**
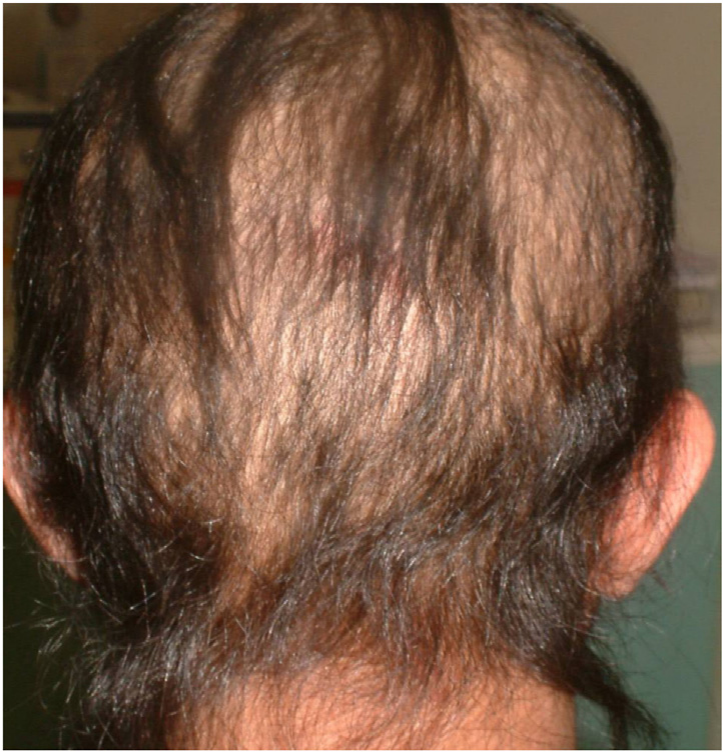
Physical examination showed a widespread and diffuse pattern of partial alopecia across all areas of the scalp.

A 32-year-old woman, presented to the dermatology outpatient clinic complaining of erythematous papules and pustules on her face, chest, and back, typical of acne vulgaris. She is a known case of HHS of the scalp due to a missense genetic mutation in lanosterol synthase, specifically [c.1172T>C; (p.Phe391Ser)] homozygous variant, on no specific treatment and reports noncompliance to topical minoxidil 5%. She had been experiencing progressive hair thinning and loss since birth. This condition had worsened after puberty, resulting in persistently sparse scalp hair with minimal growth and reports to have never had to cut her hair. A scalp examination showed rigid, coarse, uniformly pigmented hair without signs of inflammation or scarring, and a widespread, diffuse form of partial alopecia across the scalp (Fig 1). The patient had no additional symptoms such as cataracts or intellectual disability. Her eyelashes, eyebrows, pubic, and axillary hair remained unaffected, suggesting a phenotype limited to scalp hair.

**Fig 2:**
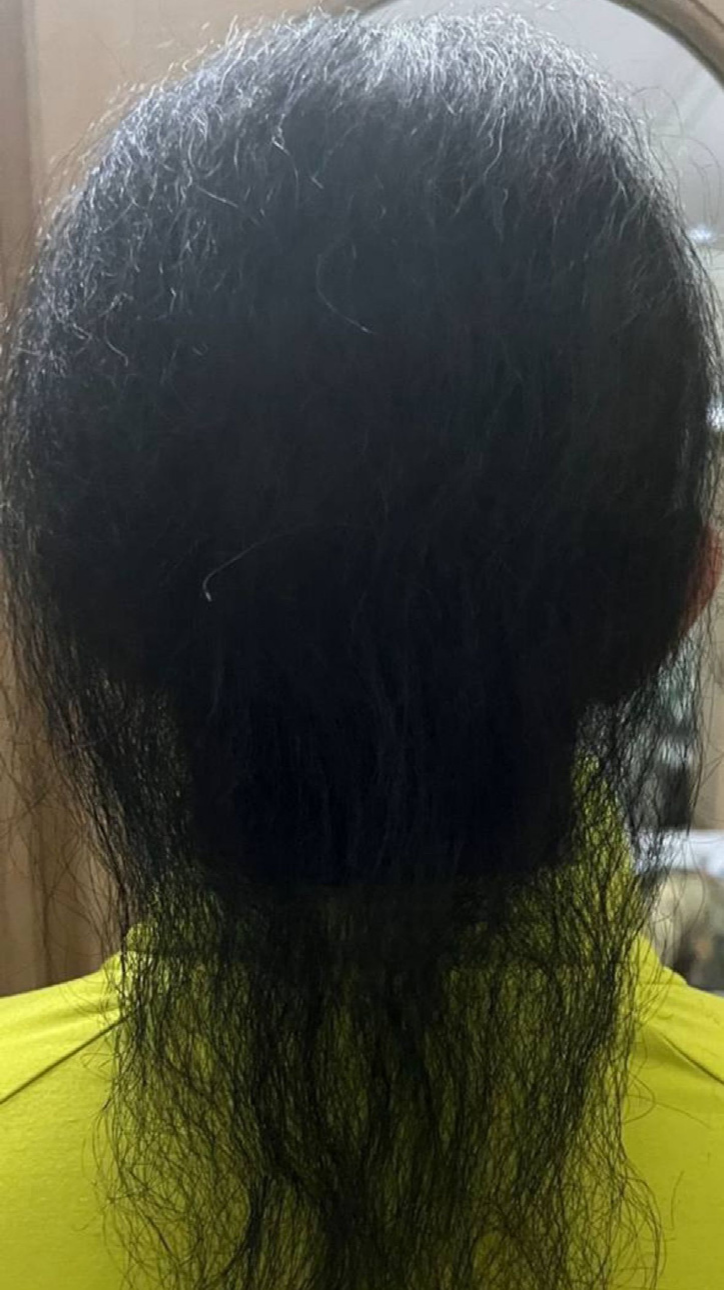
After approximately 9 months from initiating isotretinoin therapy, the patient displayed signs of hair growth on the scalp, alongside enhancements in hair length and texture.

**Fig 3:**
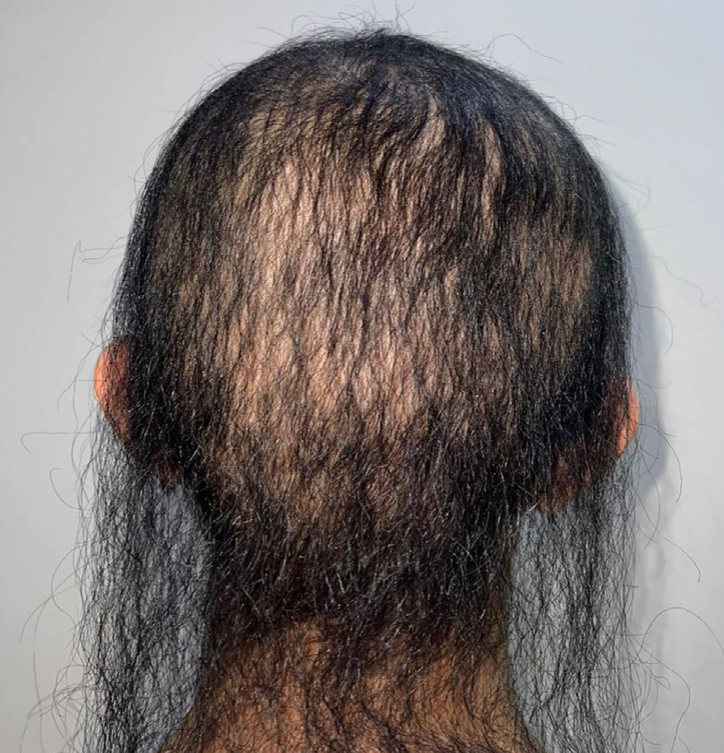
Two months after stopping isotretinoin therapy, the patient observed regression and shedding of the newly grown scalp hair.

**Fig 4:**
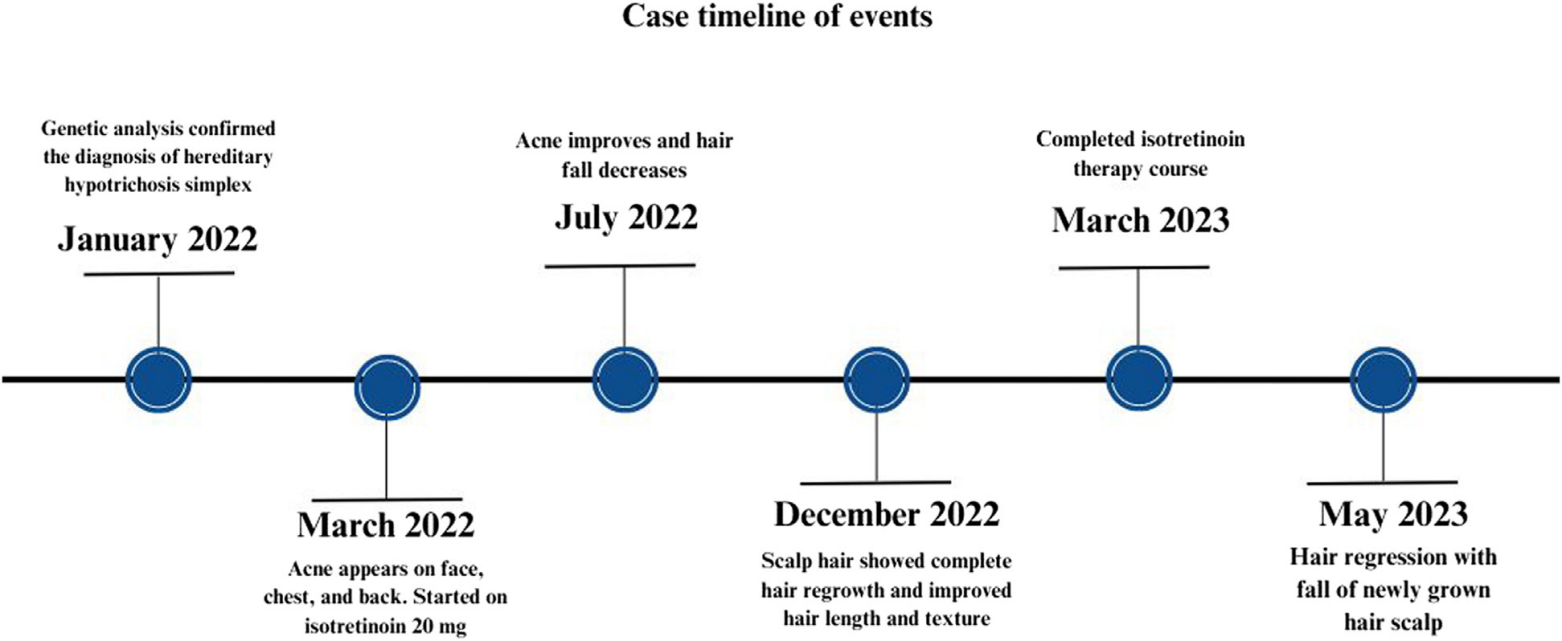
Case timeline of events.

Given her current complaint of acne lesions, she was prescribed oral isotretinoin at a dose of 20 mg. Four months after starting therapy, her acne had improved, and she reported a mild reduction in hair fall, despite reporting the nonuse of minoxidil 5% since starting isotretinoin therapy. Remarkably, about 9 months into her isotretinoin therapy for acne, she reported a significant reduction in hair fall. Even more surprising, signs of scalp hair growth and improvements in hair length and texture were observed (Fig 2). This improvement continued over time, reaching its peak after 12 months of isotretinoin therapy. However, upon discontinuation of isotretinoin, the patient experienced regression and shedding, with the newly grown scalp hair falling out after only 2 months, indicating the transient effect of the treatment (Fig 3). Case events are provided in a timeline format in (Fig 4).

## Discussion

HHS is a rare genetic nonsyndromic hair disorder characterized by alopecia either isolated to the scalp or affecting the whole body. Onset of the condition typically occurs within the first decade of life and typically leads to complete baldness by the third decade.^1^ This genodermatosis can be either autosomal dominant or autosomal recessive. In nearly 50% of cases, the specific genetic cause remains unidentified, despite the association of numerous gene mutations. Dominant forms of the disorder are correlated with mutations in CDSN, APCDD1, or SNRPE, whereas recessive forms are associated with biallelic mutations in lanosterol synthase, KRT25, LPAR6, LIPH, or DSG4.^4^ Despite its recognition for decades, effective treatment options for HHS have been limited, leaving patients with few therapeutic avenues. Recent studies have explored various interventions for HHS, offering promising outcomes. These include the use of topical, sublingual, and oral minoxidil, topical gentamicin, and platelet-rich plasma injection combined with topical minoxidil.^2^, ^3^, ^4^, ^5^, ^6^ While these treatments have shown varying degrees of success, the quest for more effective and reliable therapeutic approaches continues. Our case exhibited a favorable and unexpected response to oral isotretinoin regarding hair growth, which raises many questions considering its notorious side effects to the hair.^7^

Isotretinoin, also known as 13-cis-retinoic acid, is a retinoid derivative derived from vitamin A. Its primary indication is for the treatment of moderate-to-severe cases of acne vulgaris.^7^ Widespread approved and off-label utilization of isotretinoin beyond acne include inflammatory skin conditions, multiple genodermatoses, skin cancer, and various other dermatological disorders, due to its anti-inflammatory, immunomodulatory, and antineoplastic properties.^9^ Interestingly, our patient reported significant hair growth while on isotretinoin therapy. This phenomenon could be attributed to isotretinoin's influence on the hair cycle, potentially extending the anagen (growth) phase and reducing telogen (resting) phase duration. The exact mechanism remains unclear, but isotretinoin may modulate hair follicle biology through retinoid receptors, promoting hair growth in conditions like HHS where follicular miniaturization predominates.^7^ This case highlights the novel and unexpected response of a patient with HHS to isotretinoin therapy, suggesting its potential off-label therapeutic benefit beyond acne management in promoting hair growth. It is crucial to note that the observed improvement in hair growth was transient, as the patient experienced regression of the newly grown scalp hair following discontinuation of isotretinoin therapy. The patient was not compliant with topical minoxidil 5% prior to starting isotretinoin therapy. Upon initiation of isotretinoin for acne, she did not resume minoxidil use. Therefore, the observed hair growth can be attributed solely to isotretinoin, eliminating confounding factors from minoxidil treatment. This strengthens the hypothesis that isotretinoin independently facilitated hair regrowth in this case. In conclusion, further studies are warranted to validate these findings and elucidate the underlying mechanisms and long-term effects of isotretinoin on hair growth. Our observations contribute to expanding our understanding of HHS and offer new insights into its management.

## Conflicts of interest

None declared.
